# ETGPDA: identification of piRNA-disease associations based on embedding transformation graph convolutional network

**DOI:** 10.1186/s12864-023-09380-8

**Published:** 2023-05-25

**Authors:** Xianghan Meng, Junliang Shang, Daohui Ge, Yi Yang, Tongdui Zhang, Jin-Xing Liu

**Affiliations:** 1 School of Computer Science, Qufu Normal University, Rizhao, 276826 China; 2 Science and Technology Innovation Service Institution of Rizhao, Rizhao, 276826 China

**Keywords:** PiRNA-disease associations prediction, Heterogeneous network, Graph convolutional network, Layer attention, Embedding transformation module

## Abstract

**Background:**

Piwi-interacting RNAs (piRNAs) have been proven to be closely associated with human diseases. The identification of the potential associations between piRNA and disease is of great significance for complex diseases. Traditional “wet experiment” is time-consuming and high-priced, predicting the piRNA-disease associations by computational methods is of great significance.

**Methods:**

In this paper, a method based on the embedding transformation graph convolution network is proposed to predict the piRNA-disease associations, named ETGPDA. Specifically, a heterogeneous network is constructed based on the similarity information of piRNA and disease, as well as the known piRNA-disease associations, which is applied to extract low-dimensional embeddings of piRNA and disease based on graph convolutional network with an attention mechanism. Furthermore, the embedding transformation module is developed for the problem of embedding space inconsistency, which is lightweighter, stronger learning ability and higher accuracy. Finally, the piRNA-disease association score is calculated by the similarity of the piRNA and disease embedding.

**Results:**

Evaluated by fivefold cross-validation, the AUC of ETGPDA achieves 0.9603, which is better than the other five selected computational models. The case studies based on Head and neck squamous cell carcinoma and Alzheimer’s disease further prove the superior performance of ETGPDA.

**Conclusions:**

Hence, the ETGPDA is an effective method for predicting the hidden piRNA-disease associations.

## Background

Piwi-interacting RNAs (piRNAs) are small non-coding RNAs, which are about 30 nucleotides in length [1]. PiRNAs usually participate in multiple biological processes, including developmental regulation, transposon silencing, epigenetic regulation, and genome rearrangement by the Piwi-subfamily of Argonaute proteins [2]. PiRNAs is essential for understanding the research of small non-coding RNA [3].

Existing research shows that piRNA is closely related to human disease, which can be regarded as the initiation and control factor of tumor propagation and spread [4]. Qi et al. have found that piRNA-14633 is highly expressed in cervical cancer tissues and cells, promoting cervical tumor growth [5]. In addition, piRNAs can influence disease progression by regulating DNA methylation, for example, piRNA-6426 expression is decreased in patients with heart failure, which can inhibit hypoxia-induced cardiomyocyte dysfunction and heart failure [6]. Identifying piRNAs related to diseases can effectively promote the diagnosis and treatment of diseases [7]. However, the traditional "wet experiment" takes a lot of time, manpower and financial resources [8]. To overcome the above problems, many computational models are proposed to predict the potential piRNA-disease associations (PDAs), which is of great significance for the assistance of biological experiments [9].

In recent years, computational models have been widely concerned because of their high computational efficiency, which can provide powerful help for traditional biological experiments. Wei et al. [10] applied a positive unlabeled learning method to predict the PDAs, namely iPiDA-PUL. The samples which were not verified by the experiment are regarded as unlabeled samples. The negative samples used for training were randomly selected from unlabeled samples, which employed a parallel random forest as the classifier to predict PDAs. Since there was a great possibility of positive correlation in unlabeled samples, which may reduce the recall rate of the classifier, so Wei et al. [11] constructed a predictor to select more reliable negative samples from unlabeled samples (iPiDA-sHN). It extracted disease features through a convolutional neural network and uses a support vector machine to predict PDAs. Ji et al. [12] proposed a method based on the deep feature learning model (DFL-PiDA), which used the convolutional denoising autoencoder depth learning to extract four types of similarity features, and a limit learning machine was used as the training model to predict potential PDAs. Zheng et al. [13] introduced the stackable automatic encoder into PDAs prediction based on multi-source information. After the features were denoised by the automatic encoder, the random forest classifier was applied to predict the potential PDAs. Qian et al. [14] constructed a calculated the Jaccard similarity of diseases model, namely iPiDA-GBNN. The iPiDA-GBNN extracted key features through a stackable automatic encoder. The known associations and negative associations were trained through the gradient-enhanced neural network to predict PDAs. Syed et al. [15] proposed a hierarchical model consisting of CNN and a full connection layer based on deep learning. Using one-hot coding was regarded as the input of CNN. The original piRNA sequence information was encoded into one-dimensional feature vectors, which were fused with the features extracted through CNN. Then high-dimensional features are extracted by the full connection layer, which makes full use of the information between piRNA and diseases and makes the algorithm more robust. Zheng et al. [16] constructed a model that added structural disturbance to the network for PDAs (SPRDA). The impact of negative samples was eliminated and it increased the structure consistency index to measure the feasibility of prediction, which has achieved high prediction performance. Zhang et al. [17] developed a model to identify the PDAs (iPiDA-LTR), which was based on learning sequencing. The iPiDA-LTR can not only identify the deletion associations between known piRNA and diseases but also detect the associations with potential PDAs. However, the above methods generally have problems of low prediction accuracy and model robustness, so many models based on the graph convolutional network are proposed for predicting PDAs.

The association information between nodes and edges in graph structure can improve the prediction accuracy for PDAs models. Therefore, Hou et al. [18] Proposed a model, which regarded the PDAs problem as a link prediction problem named iPiDA-GCN and generated node information by restarting random walks. Two GCN models were constructed to further capture the node embedding of the network. Zheng et al. [19] developed a model based on the line graph attention network for the prediction of PDAs. The features of the node itself and adjacent nodes were fused through the network, which improved the information coverage. In addition, the feed-forward neural network was applied to map the features to real numbers for predicting the PDAs score, which turned link prediction into node prediction. In general, the above models based on GCN show their advantages in predicting PDAs from different perspectives.

**Fig. 1:**
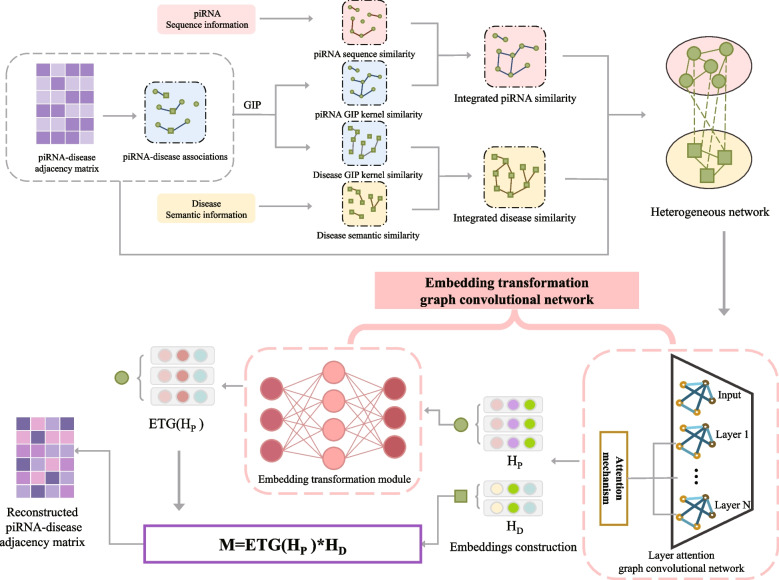
The flow chart of ETGPDA

In this study, a method based on embedding transformation graph convolutional network was proposed for predicting hidden PDAs (ETGPDA). The flow chart of this model is shown in Fig. 1. Specifically, a heterogeneous network is firstly constructed by the integrating similarity information of piRNAs and diseases and the known PDA informations, which are applied to extract low-dimensional embeddings of piRNA and disease based on GCN with an attention mechanism. Then, the embedding transformation module is developed to covert piRNA and disease embeddings into the same space. Finally, cosine similarity is employed to obtain the PDA score. The same space conversion function of different embeddings of embedding transformation module greatly improves the robustness and performance of ETGPDA. The results of the AUC based on five-fold cross-validation show that the ETGPDA was better than the other five selected computational models. Furthermore, the case studies based on Head and neck squamous cell carcinoma and Alzheimer’s disease further prove the superior performance of ETGPDA.

## Materials and methods

### Human piRNA-disease associations

The piRDisease v1.0 database [20] contains 7939 experimentally verified PDAs. After removing duplicate associations and non-human piRNAs, 5002 experimentally verified PDAs between 4350 piRNAs and 21 diseases were determined, which are defined as follows:

1 $$S={S}^{P}\bigcup {S}^{U}$$

where ${S}^{P}$ represents 5002 known PDAs, which is a set of positive associations, and ${S}^{U}$ represents a set of 86,348 unknown PDAs. The association matrix ${A\in {\mathbb{R}}}^{M\times N}$ is used to represent the known PDAs, where $M$ and $N$ represent the number of piRNA and diseases, respectively.

2 $$A\left({p}_{a},{d}_{b}\right)=\left\{\begin{array}{c}1, \left({p}_{a},{d}_{b}\right) \in {S}^{P}\\ 0, \left({p}_{a},{d}_{b}\right) \in {S}^{U}\end{array}\right.$$

where ${(p}_{a},{p}_{b})$ represents the associations between the ${a}^{th}$ piRNA and the ${b}^{th}$ disease, if they have known association, $A{(p}_{a},{d}_{b})$ is set to 1, otherwise, it is 0.

### PiRNA similarity

The piRBase v2.0 [21, 22] database contains the sequence information of piRNA, which is used to calculate the piRNA sequences similarity score based on the Needleman-Wunsch algorithm [23], which is represented as matrix $SP\in {P}^{M\times M}$ and $M$ denotes the number of piRNAs. To handle the randomness of the similarity score, a standardized operation is selected and shown in formula (3), ${P}_{S}{(p}_{a},{p}_{b})$ is the processed piRNA sequence similarity score.

3 $$P_S\left(p_a,p_b\right)=\frac{P_S\left(p_a,p_b\right)-P_S^{min}}{P_S^{max}-P_S^{min}}$$

where $P_S^{min}$ and $P_S^{max}$represent the minimum and the maximum similarity score in the sequence similarity matrix $SP$, respectively. The final standardized matrix is shown as follows:

4 $$SP \left({p}_{a},{p}_{b}\right)=\left\{\begin{array}{ll}1,&{p}_{a}={p}_{b}\\{P}_{S}\left({p}_{a},{p}_{b}\right),&p_a\ne{p_b}\end{array}\right.$$

Gaussian interaction profile (GIP) kernel similarity is a common collaborative filtering algorithm [24–26], which is a common similarity measurement method in ncRNA-disease associations prediction. Based on the association matrix $A$, the piRNA GIP similarity matrix can be obtained by formula (5):

5 $$G_p\left(p_a,\;p_b\right)=\exp(-\varphi_p\parallel V\left(p_a\right)-V\left(p_b\right)\parallel^2)$$

where $V({P}_{a})$ represents the row vector between piRNA a and 21 diseases, and $V({P}_{b})$ represents row vector between piRNA b and 21 diseases, ${\varphi }_{p}$ represents the parameters that control the bandwidth of the original core. The definition is shown as formula (6):

6 $${\varphi }_{p}=\frac{1}{\frac{1}{{num}_{p}}\sum_{k=1}^{{num}_{p}}\parallel V({p}_{k}){\parallel }^{2}}$$

where ${num}_{p}$ represents the number of piRNAs.

To handle that the single similarity information cannot provide sufficient prior information, piRNA sequence similarity is integrated with GIP similarity. The formulas for the integrating similarity of piRNA is as follows:

7 $$SPG\left({p}_{a},{p}_{b}\right)=\left\{\begin{array}{cc}P_s\left(p_a,p_b\right),&{p}_{a}\,and\,{p}_{b}\,have\,sequence \,similarity\\{G}_{p}\left({p}_{a},{p}_{b}\right),&otherwise\end{array}\right.$$

### Disease similarity

**Fig. 2:**
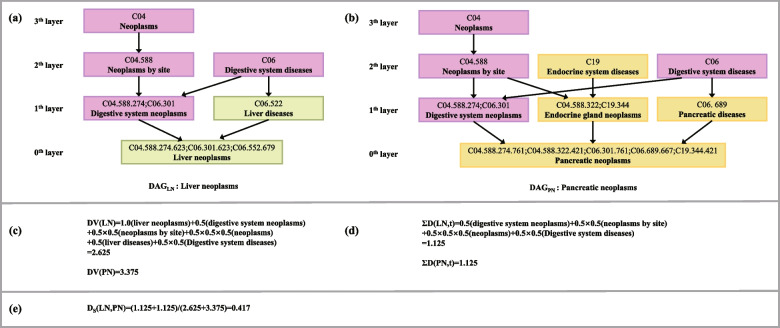
DAG representation of disease liver neoplasms and pancreatic neoplasms

Disease semantic similarity score can be calculated by a directed acyclic graph of disease (DAG) [25], which is obtained by the $MesH$ database (https://www.nlm.nih.gov/). It provides DAG information about all diseases [27]. Inspired by the literature [28], the disease semantic similarity score is calculated and denoted as a matrix $SD$. Take disease liver neoplasms $(LN)$ and pancreatic neoplasms $(PN)$ as an example, the specific calculation process is described as follows. The DAGs of both are shown in Fig. 2, where nodes denote a specific disease $MesH$ descriptor.

In Fig. 2 (a) and (b), the node of layer 0 represents the $MesH$ descriptor of $LN$ and $PN$, respectively and their semantic contribution value is 1. The higher the number of layers, the smaller the semantic contribution of this node to the disease $LN$ and $PN$, so the semantic contribution factor is introduced here to control the semantic contribution of the disease nodes in different layers, which is defined as 0.5 confirmed by literature [28]. The semantic contribution values $DV(LN)$ and $DV(PN)$ of disease $LN$ and $PN$ are shown in Fig. 2 (c) [29].

Based on the semantic contribution value of the two diseases, ${T}_{LN}$ and ${T}_{PN}$ are defined as a nodeset, which contains all the ancestor nodes of a node $LN$ or their own. In addition, $t\in {T}_{LN}\cap {T}_{LN}$ represents the common node of the DAG graph of two diseases, which semantic contribution values are shown in Fig. 2 (d). Therefore, the semantic similarity ${D}_{S}(LN,PN)$ between disease $LN$ and $PN$ is calculated in Fig. 2 (e). The disease similarity matrix calculated above is shown as follows:

8 $$SD\left({d}_{a},{d}_{b}\right)=\left\{\begin{array}{ll}1,&d_a=d_b\\D_s\left(d_a,d_b\right),&d_a\ne{d_b}\end{array}\right.$$

Similar to piRNA, ${G}_{d}\left({d}_{a},{d}_{b}\right)$ represents the GIP similarity between different diseases, which is shown in formula (9):

9 $${G}_{d}\left({d}_{a},{d}_{b}\right)=\mathrm{exp}(-{\varphi }_{d}\parallel V\left({d}_{a}\right)-V({d}_{b}){\parallel }^{2})$$

where $V({d}_{a})$ denotes the correlation vector between disease a and 4350 piRNAs and $V({d}_{b})$ represents the correlation vector between disease b and 4350 piRNAs. Similarly, the definition of ${\varphi }_{d}$ is shown in formula (10):

10 $${\varphi }_{d}=\frac1{\frac1{{num}_d}\sum_{k=1}^{{num}_d}\parallel V(p_k)\parallel^2}$$

where ${num}_{d}$ represents the number of diseases.

Similar to piRNA, disease semantic similarity is integrated with GIP similarity. The formula for the integrating similarity of disease is as follows:

11 $$SDG\left(d_a,d_b\right)=\left\{\begin{array}{cc}D_s\left(d_a,d_b\right),&d_a\;and\,d_b\,have\,semantic\,similarity\\G_d\left(d_a,d_b\right),&otherwise\end{array}\right.$$

### Embedding transformation graph convolutional network (ETGPDA)

In this study, a model (ETGPDA) was proposed based on embedding transformation graph convolutional network to predict potential PDAs, which is mainly divided into the following parts: (1) A heterogeneous network is constructed based on the integrated similarity of piRNA, disease and the known PDAs; (2) Heterogeneous network is applied to the GCN based on embedding transformation to extract the low-dimensional embeddings of piRNA and disease. (3) Cosine similarity calculation is used to predict the final potential PDAs.

### Construction of the heterogeneous network

Firstly, the integrated similarities are normalized by eliminating the randomness of network edge weight. The calculation formula is as follows:

12 $$NSPG={D}_{p}^{-\frac{1}{2}}SPG({p}_{a},{p}_{b}){D}_{p}^{-\frac{1}{2}}$$

13 $$NSDG={D}_{d}^{-\frac{1}{2}}SDG({d}_{a},{d}_{b}){D}_{d}^{-\frac{1}{2}}$$

where

14 $${D}_{P}=diag\left({\sum }_{pb}SPG({p}_{a},{p}_{b})\right)$$

15 $${D}_{d}=diag\left({\sum }_{{d}_{b}}SDG\left({d}_{a},{d}_{b}\right)\right)$$

Then, the two-layer heterogeneous network is constructed based on piRNA integrating similarity $SPG\left({p}_{a},{p}_{b}\right)$, disease integrating similarity $SDG\left({d}_{a},{d}_{b}\right)$ and piRNA-disease association matrix $A\left({p}_{a},{d}_{a}\right)$, which is represented as follows:

16 $${A}_{H}=\left[\begin{array}{cc}NSPG& A\\ {A}^{T}& NSDG\end{array}\right]$$

### Graph convolutional network

In ETGPDA, GCN is applied to extract the low-dimensional embeddings of piRNA and disease [30]. The heterogeneous network ${A}_{H}$ contains not only the link information of nodes but also the information of nodes themselves. Therefore, the penalty factor $\omega$ of heterogeneous networks is set to control the contribution of similarity in the GCN propagation process, and the input graph $B$ is shown as follows:

17 $$B=\left[\begin{array}{cc}\omega NSPG& A\\ {A}^{T}& \omega NSDG\end{array}\right]$$

In GCN, the node embeddings of the layer $L$ are treated as the input of layer $L+1$ to extract low-dimensional embeddings. We first initialize the embedding ${H}^{(0)}$, which is shown as:

18 $${H}^{(0)}=\left[\begin{array}{cc}0& A\\ {A}^{T}& 0\end{array}\right]$$

After the above steps, the node embedding ${H}^{(1)}$ of the first layer of GCN is obtained, which is shown in formula (19).

19 $${H}^{(1)}=f\left({D}^{-\frac{1}{2}}{BD}^{-\frac{1}{2}}{H}^{(0)}{W}^{(0)}\right)$$

where $f(x)$ is the non-linear activation function ReLU and ${W}^{(0)}$ represents the weight matrix between the input layer and the hidden layer. Node embedding ${H}^{(L)}$ of the GCN layer $L$ is obtained through forward propagation.

20 $${H}^{(L)}=f\left({D}^{-\frac{1}{2}}{BD}^{-\frac{1}{2}}{H}^{(L-1)}{W}^{(L-1)}\right)$$

where ${H}^{(L-1)}$ is the embedding of piRNA and disease nodes in a heterogeneous network, and ${W}^{(L-1)}$ is the weight matrix between the $L-1$ layer and the $L$ layer. K-dimensional embeddings are obtained from different convolution layers after L times forward propagation.

The known and unknown PDAs are regarded as the positive correlation subset $Y+$ and the negative correlation subset $Y-$, respectively. However, 5002 positive association subsets and 86,348 negative association subsets, can affect the calculation of most losses, which are difficult to provide useful information. To solve the problem of unbalanced positive and negative samples, we choose the weighted cross-entropy loss function [31], which is shown as follows:

21 $$Loss=-\frac{1}{N\times M}\left(\mu \times {\sum }_{\left(i,j\right)\in Y+}\mathrm{log}{a}_{ij}^{^{\prime}}+{\sum }_{\left(i.j\right)\in Y-}\mathrm{log}(1-{a}_{ij}^{^{\prime}})\right)$$

where $\mu =\frac{\left|Y-\right|}{\left|Y+\right|}$, which reduces the impact of sample imbalance by emphasizing the importance of positively correlated subsets, $\left|Y-\right|$ represents the number of samples in negative subsets, and $\left|Y+\right|$ represents the number of samples in positive subsets.

The Adam optimizer [32] is selected to optimize the weighted cross-entropy loss function, and the loss is minimized through the back-propagation algorithm. A large number of parameters lead to over-fitting or some neurons not being activated during training. We introduce the dropout technology [33] into the convolution layer, which is to randomly discard a part of neurons and their connected edges during the training process. It is regarded to divide ETGPDA into several small models, which are trained on different subnets and integrated to predict [34]. Dropout technology improves regularization methods and can effectively prevent over-fitting [35].

To better balance the training speed, the cyclic learning rate is introduced, which changes the learning rate between the maximum and the minimum and effectively improves the accuracy of the model [36].

In GCN, different convolution layers capture different structural embeddings from heterogeneous networks [37]. Specifically, the first layer captures the direct link information of the current node, and the second captures its two-hop neighbor information. The higher layer captures the multi-hop neighbor information through an iterative update [38]. Since neighbor information at different distances has different effects on nodes, the embeddings extracted from different convolution layers in GCN have different contributions to each node, so we introduce an attention mechanism to solve this problem. It is shown as follows:

22 $$H={a}_{1}{H}^{(1)}+{a}_{2}{H}^{(2)}+\dots +{a}_{L}{H}^{(L)}$$

Where ${a}_{L}$ is the weight of different layers obtained through convolutional network learning. We initialize it as:

23 $$\begin{array}{cc}{a}_{L}=\frac{1}{L+1},& L=\mathrm{1,2},3\dots \end{array}$$

After weighted sum calculation, the final k-dimension embedding of ETGPDA is obtained:

24 $$F=\left[\begin{array}{c}{H}_{P}\\ {H}_{D}\end{array}\right]$$

where, ${H}_{P}$ and ${H}_{D}$ represent the embeddings of piRNA and disease, respectively. The final k-dimension embedding is input into the embedding transformation module.

### Embedding transformation module

Due to the low similarity between, ${H}_{P}$ and${H}_{D}$, the learnable weight matrix is constructed based on an existing solution to calculate the embedding similarity, which is essentially a transformation of the polar coordinate system and cannot change the space of the embedding when performing linear operations on the matrix. At the same time, the large amount of learning parameters determines that the embedding dimension cannot be too small in the linear model, which leads to the amount of training increase.

To solve the above problems, an embedding transformation module is developed to transform the embedding representation of piRNA and disease into the same embedding space, which is a four-layer convolutional neural network, including an input layer, hidden layer, and output layer. ${H}_{P}$ is the input of the embedding transformation network, which passes through layer1, layer2, and finally outputs the transformed matrix $ETG$(${H}_{P}$). To make ETG(${H}_{P}$) to be close enough to ${H}_{D}$, the embedding dimensions of layer 1 and layer 2 are set to be twice and quadruple the input layer, respectively. The analysis of parameter quantity is shown in Table 1, which can be seen that the embedding transformation module can effectively reduce the number of parameters of the model, and can learn the lower-dimensional embedding representation. At the same time, the non-linear activation function $ReLU$ is set at each layer, which makes the transformation process no longer a simple linear transformation process, and greatly improves the learning ability of the model.

**Table 1:** Analysis of parameter quantity

Method	Param (Kb)
With Hide Layer	3.50
Without Hide Layer	68.29

Finally, we calculate the similarity between $ETG({H}_{P})$ and ${H}_{D}$ through cosine similarity operation [39], and the similarity matrix $M$ represents the final predicted PDA matrix:

25 $$M=ETG\left(H_P\right)\;\cdot\;H_D^T$$

## Results

### Performance evaluation

In this study, the prediction performance of the ETGPDA is tested through the fivefold cross-validation (FFCV). We randomly divided all the known PDAs into five groups and selected one group in turn as the test data set, and the other four groups as the training data set, which takes 10 times to average the AUC, accuracy, recall, and specificity. The AUC applies to performance analysis in unbalanced data sets. The Accuracy indicates the correct proportion of the predicted positive and negative samples. Recall denotes the probability of the predicted positive sample in the known positive sample. The specificity represents the proportion of predicted negative samples to all negative samples. A value closer to 1 indicates better performance for the model.

### Performance comparison

**Fig. 3:**
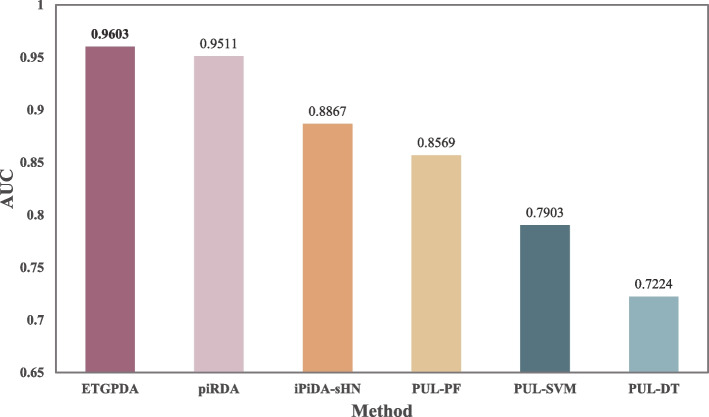
Comparison of AUC values of ETGPDA and other methods with FFCV

We compare the ETGPDA with other five models for PDAs prediction, including piRDA [15], iPiDA-sHN [11], PUL-PF [10], PUL-SVM [10], and PUL-DT [10], to prove that ETGPDA has superior performance. The comparison results of AUC values are shown in Fig. 3, which can be seen that the AUC values of piRDA, iPiDA-sHN, PUL-PF, PUL-SVM, and PUL-DT are 0.9511, 0.8867, 0.8569, 0.7903, and 0.7224, respectively. The AUC value of the ETGPDA is 0.9603, which is higher than the other five models.

### Effect of parameters

**Fig. 4:**
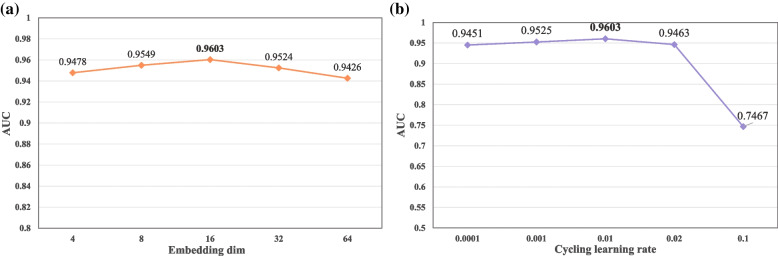
**a** AUC values of ETGPDA depending on the different embedding dimension parameter values; **b** AUC values of ETGPDA depending on the different cycling learning rate parameter values

FFCV is employed to explore the impact of embedding dimension and cycling learning rate on the ETGPDA, in which the embedding dimension is set to 4, 8, 16, 32, and 64 respectively and the cycling learning rate is set to 0.0001, 0.001, 0.01, 0.02 and 0.1 respectively. Figure 4 (a) and (b) show the AUC values of embedded dim and cycling learning rates with different parameter values, respectively. It can be seen that the performance of the ETGPDA is the best when the value of the embedding dimension is 16 and the value of the Cycling learning rate is 0.01. At this time, the parameter quantity of ETGPDA is 3.5 kb, which also shows that the efficiency of the model is high.

### Impact of dropout technique on ETGPDA

**Fig. 5:**
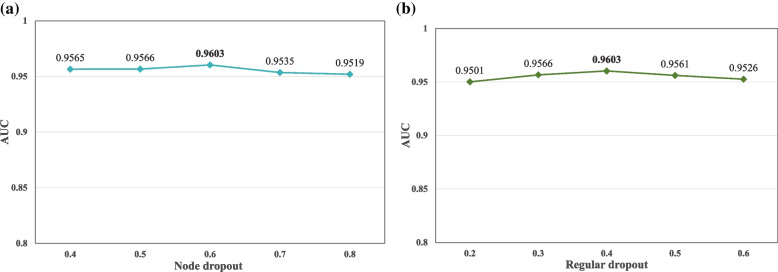
**a **AUC values of ETGPDA depending on the different node dropout values; **b** AUC values of ETGPDA depending on the different regular dropout values

A large amount of parameters and a lack of train samples can lead to the over-fitting phenomenon in GCN training, which will affect the performance of ETGPDA. Therefore, node dropout and regular dropout are selected to eliminate these defects. In the process of forward propagation, the model will not rely too much on some local features by letting the activation value of a neuron stop working with a certain probability. The values of node dropout are set to 0.4, 0.5, 0.6, 0.7, and 0.8 respectively and regular dropout are set to 0.2, 0.3, 0.4, 0.5, and 0.6 respectively, which are shown in Fig. 5 (a) and (b). It can be seen that the performance of the ETGPDA is the best when the node dropout value and the regular dropout value are 0.6 and 0.4, respectively, which indicates ETGPDA has a strong generalization ability.

### Impact of non-linear layer on ETGPDA

To identify the influence of the non-linear layer on ETGPDA, an ablation experiment is carried out for the embedding transformation module. A linear model is constructed by removing the non-linear activation function $ReLU$. The experimental results of AUC, accuracy, recall, and specificity are presented in Table 2, which can be seen as the performance of ETGPDA is worse when linear functions are applied as decoders. Therefore, a non-linear layer is employed to improve the learning ability of ETGPDA, which contains a large number of non-linear activation functions.

**Table 2:** Performance comparison between ETGPDA and removing activation function $ReLU$

Testing set	AUC↑	Accuracy↑	Recall↑	Specificity↑
With Activation Function	**0.9603**	**0.9551**	**0.8636**	**0.9565**
Without Activation Function	0.9066	0.9397	0.8434	0.9309

### Impact of attention mechanism on ETGPDA

To identify the influence of the attention mechanism on ETGPDA, an ablation experiment is carried out. Specifically, the embeddings of each convolution layer are added without an attention mechanism. The experimental results of the AUC, accuracy, recall, and specificity are presented in Table 3, and which can be seen that if the contribution of different convolution embeddings to nodes is equal, the prediction accuracy will be reduced. Therefore, the attention mechanism is introduced to assign different weights to each convolution layer. The final embedding is the weighted sum of the embeddings of different convolution layers, which effectively improves the prediction accuracy of ETGPDA.

**Table 3:** Performance comparison between ETGPDA and removing attention mechanism

Testing set	AUC↑	Accuracy↑	Recall↑	Specificity↑
With Attention Mechanism	**0.9603**	**0.9551**	**0.8636**	**0.9565**
Without Attention Mechanism	0.9506	0.9450	0.8308	0.9464

### Case study

To further demonstrate the prediction performance, two important diseases: Head and neck squamous cell carcinoma, and Alzheimer’s disease are selected to predict PDAs. Head and neck squamous cell carcinoma is epithelial cancer that occurs in the mouth, throat, and other parts, which is the sixth most common cancer in the world and is mainly affected by smoking, drinking, and other factors [40]. Alzheimer’s disease mostly occurs in the elderly over 65 years old. There are at least 50 million dementia patients in the world at present, which is expected to reach 152 million by 2050, of which about 60%-70% are Alzheimer's disease patients [41]. Therefore, the study of these two diseases is of great significance to human health.

The top-ten piRNAs of the two diseases were obtained by descending the correlation scores predicted by ETGPDA. The selected 20 PDAs were analyzed in detail, and the relevant literature was searched. The results are shown in Table 4. It can be seen that most PDAs have been verified by biological experiments and the relevant biological literature. Therefore, ETGPDA has good prediction performance.

**Table 4:** ETGPDA predicts the top-ten related piRNAs of two important diseases

Disease	piRNA	Associated score	Evidence
Head and neck squamous cell carcinoma	piR-hsa-28394 piR-hsa-28395 piR-hsa-23992 piR-hsa-27493 piR-hsa-23209 piR-hsa-15399 piR-hsa-23210 piR-hsa-1823 piR-hsa-1282 piR-hsa-5937	0.902 0.887 0.885 0.863 0.853 0.837 0.826 0.775 0.732 0.730	PMID:28,109,471 PMID:28,109,471 PMID:27,323,410 PMID:27,323,410 PMID:28,109,471 Unconfirmed PMID:28,109,471 Unconfirmed Unconfirmed PMID:28,109,471
Alzheimer’s disease	piR-hsa-23210 piR-hsa-1849 piR-hsa-23209 piR-hsa-20266 piR-hsa-1823 piR-hsa-20266 piR-hsa-15023 piR-hsa-1191 piR-hsa-31236 piR-hsa-18287	0.858 0.856 0.849 0.830 0.827 0.806 0.799 0.773 0.685 0.603	PMID:28,127,595 PMID:28,127,595 PMID:28,127,595 Unconfirmed PMID:28,127,595 Unconfirmed PMID:28,127,595 PMID:28,127,595 Unconfirmed Unconfirmed

## Discussion

Based on the assumption that similar piRNAs are often associated with the same disease, a method based on embedding transformation graph convolutional network was proposed for predicting hidden PDAs (ETGPDA), which is confirmed to be superior to the other five methods through FFCV. Highlights of the ETGPDA lie in the embedding transformation module, which ensures learning ability and prediction accuracy.

Currently, most of the methods for associations prediction of non-coding RNAs, such as miRNA, lncRNA, piRNA and so on, with diseases pay attention to the presence or absence of associations. However, deeper studies, such as association types, up- and down-regulation relationships and reciprocal association relationships, are rare. In the future, we will consider integrating more perspectives of piRNA and disease similarity information aiming to provide enough priori information for ETGPDA. In addition, the introduction of deep learning models such as relational graph attention networks for the indepth study of PDAs to better find the types and causes of piRNAs that cause complex diseases, which can be a powerful aid for biological experiments. Of course, the impact of piRNA interaction on specific diseases will be considered, which has important reference value for the study of human disease prevention, diagnosis, and treatment.

## Conclusions

In this study, a method based on embedding transformation graph convolutional network was proposed for predicting hidden PDAs embedding transformation module. Specifically, a two-layer heterogeneous network is firstly constructed by the integrating similarity information of piRNAs and diseases and the known PDA informations, which are applied to extract low-dimensional embeddings of piRNA and disease based on GCN with an attention mechanism. Then, the embedding transformation module is developed to covert piRNA and disease embeddings into the same space. Finally, cosine similarity is employed to obtain the PDA score. The same space conversion function of different embeddings of embedding transformation module greatly improves the robustness and performance of ETGPDA. The results of the AUC based on five-fold cross-validation show that the ETGPDA was better than the other five selected computational models. Furthermore, the case studies based on Head and neck squamous cell carcinoma and Alzheimer’s disease further prove the superior performance of ETGPDA.

The reasons for the superior predictive performance of ETGPDA are summarized below: (1) Integrates data information from multiple sources, making the data more comprehensive. (2) Using GCN to extract embeddings improves the robustness of the model. (3) The proposed embedding transformation module transforms the embedding space, making the model more lightweight and greatly improving the learning capability. However, it still has some defects. Firstly, it cannot eliminate the dependence on known PDAs. In addition, the sparsity of raw data has a great impact on the prediction performance of ETGPDA. In the future, we intend to continue to optimize the predictive performance of the PDAs model and to conduct in-depth studies on the up- and down-regulation of piRNAs with diseases and the different association types of PDAs, aiming to provide a powerful aid for biological experiments.

## Data Availability

The ETGPDA is implemented in Python. Its source code, user manual and related experimental data are available online at https://github.com/CDMB-lab/ETGPDA .
